# The relationship between physical activity level, attitude to seek psychological help, and mental development in adolescents

**DOI:** 10.3389/fpsyg.2026.1774136

**Published:** 2026-03-25

**Authors:** Oğuzhan Çalı, Rahmi Yıldız

**Affiliations:** Faculty of Sports Sciences, Sivas Cumhuriyet University, Sivas, Türkiye

**Keywords:** adolescence, attitudes toward seeking psychological help, mental health, mental development, physical activity

## Abstract

**Introduction:**

Psychological health problems during adolescence affect both individual well-being and society. Although physical activity supports mental health, adolescents’ attitudes toward seeking psychological help are also essential. This study examined the relationships between physical activity level, help-seeking attitudes, and mental development in adolescents.

**Methods:**

A descriptive survey design was conducted with 520 adolescents aged 12–18 years attending middle and high schools in Sivas, Türkiye. Data were collected using the International Physical Activity Questionnaire–Short Form, the Attitudes Toward Seeking Psychological Help Scale, and the Mental Development in Adolescence Scale. Descriptive statistics, Pearson correlation analysis, and simple linear regression analysis were applied.

**Results:**

Very strong positive correlations were found between the total help-seeking attitude score and its positive attitude subdimension across all physical activity levels (inactive *r* = 0.837; active *r* = 0.887; highly active *r* = 0.911; all *p* < 0.001). Moderate positive correlations were observed between total help-seeking and negative attitudes (*r* = 0.482–0.577, *p* < 0.001). Mental development showed strong positive associations with its subdimensions regardless of activity level, particularly Personal Lack of Control (*r* = 0.904–0.906, *p* < 0.001) and Shyness (*r* = 0.626–0.766, *p* < 0.001). Associations between help-seeking attitudes and mental development were weak; however, negative help-seeking attitudes were significantly and positively related to Personal Lack of Control (*r* = 0.318, *p* < 0.01) and total mental development (*r* = 0.290, *p* < 0.01).

**Conclusion:**

Adolescents’ help-seeking attitudes and mental development appear multidimensional and are shaped mainly by psychosocial factors rather than physical activity alone, highlighting the need to integrate psychosocial components into adolescent mental health interventions.

## 1 Introduction

Mental health problems emerging during adolescence and young adulthood are identified by the World Health Organization as major determinants of morbidity and mortality, with particular emphasis placed on the widespread neglect of this issue (Gore et al., 2011). This developmental period is characterized by rapid biological, cognitive, interpersonal, and environmental changes, as well as increased exposure to stressful life events, rendering adolescents more vulnerable to psychological disorders (Blakemore, 2019). A substantial proportion of these mental health difficulties persist into adulthood, and it has been observed that even adolescents experiencing severe psychological distress may avoid seeking help (Mori et al., 2024). Untreated mental health problems impair daily functioning and often extend into later stages of life (Patel et al., 2018). The importance of youth mental health extends beyond individual well-being and has significant implications for the social and economic structures of societies (Meherali et al., 2021).

Regular physical activity is widely recognized not only for its contribution to physical health, but also for reducing mortality risk and improving both longevity and quality of life (Westcott, 2012; Gill et al., 2013; Xu et al., 2025). Studies among university students consistently demonstrate an inverse relationship between regular physical activity participation and symptoms of depression, anxiety, and stress, along with improvements in overall quality of life (Ghrouz et al., 2019; Pengpid and Peltzer, 2020; Herbert et al., 2020). Similarly, research in adolescent populations indicates that physical activity serves as a potential buffer against daily stress (Flueckiger et al., 2016). In another study, physical activity was found to exert positive effects on general psychological well-being and quality of life, reducing stress levels, enhancing mood, and alleviating depressive symptom severity. Furthermore, positive affect experienced during physical activity appeared to play a protective and reinforcing role in psychological well-being and resilience (Altın et al., 2025). Complementary findings also suggest that regular physical activity not only improves physical and mental health in adolescents but also enhances social well-being, reduces chronic disease risk factors, and contributes to higher self-esteem and academic performance (Tambalis, 2022).

Help-seeking behaviors among adolescents are of critical importance for alleviating psychological distress and facilitating personal growth. School-based psychological counseling is considered an effective approach for promoting students’ mental health and emotional well-being (Cooper, 2013; Toth et al., 2024). However, many adolescents avoid mental health services despite their needs and instead turn to non-professional sources of support (Schnyder et al., 2020; Nguyen Thai and Nguyen, 2018). Factors such as fear of stigma, family beliefs, mental health literacy, and autonomy influence this behavior (Aguirre-Velasco et al., 2020). Structural barriers—including limited-service availability, financial constraints, transportation challenges, and long waiting periods—also restrict access to psychological help (Aguirre-Velasco et al., 2020). Moreover, confidence in mental health professionals and the perceived quality of the therapeutic relationship may either facilitate or hinder help-seeking processes (Reardon et al., 2017).

Despite recognizing symptoms of depression and stress, adolescents often perceive professional services as insufficiently helpful and prefer seeking support from their social networks (Nguyen Thai and Nguyen, 2018). These findings highlight the influence of individual, cultural, and structural barriers on professional help-seeking behaviors. Approximately 20% of children and adolescents are known to experience clinically significant psychological problems, and about 50% of adult mental disorders originate during adolescence (Belfer, 2008). Early intervention therefore plays a critical role in reducing both current and future psychopathology.

Recent studies further support the protective role of physical activity. Rodríguez-Romo et al. (2023) reported that increasing physical activity levels were associated with reduced mental health problems among university students, with high levels of leisure-time physical activity yielding the strongest protective effect (47% lower risk). Mahindru et al. (2023) demonstrated that regular physical activity and yoga reduce negative symptoms in schizophrenia, help manage cravings in alcohol dependence, alleviate symptoms of depression and anxiety, and improve sleep quality. In a study conducted in Chile with 351 high school students, Merellano-Navarro et al. (2025) found high prevalence rates of depression (54.4%), anxiety (63%), and stress (42.2%), with low physical activity levels, poor parental relationships, and low self-concept/motivation significantly associated with these risks.

Recent epidemiological studies conducted in Türkiye further corroborate these findings while also delineating the country-specific magnitude of the problem. Approximately 81% of adolescents do not meet recommended levels of physical activity (Çakir et al., 2025). Moreover, a large-scale study among university students demonstrated that higher levels of physical activity were significantly associated with increased psychological resilience, self-confidence, and self-efficacy (Fidan and Yıldırım, 2025). Similarly, Doğan Ustun and Erkal (2025) reported that the purpose of sport participation (e.g., health/recreation versus amateur athletic involvement) was linked to significant differences in mental toughness and stress-coping strategies. Importantly, the type and contextual characteristics of physical activity were also identified as determining factors in this relationship. Collectively, these findings indicate that the protective effect of physical activity on mental health is also evident within Turkish samples and underscore the necessity of implementing interventions aimed at promoting physical activity, particularly among young populations.

This study makes several novel contributions to the literature on adolescent mental health, physical activity, and help-seeking behavior. Unlike prior research that has typically examined these constructs in isolation, the present study adopts an integrated analytical model, simultaneously investigating physical activity alongside both positive and negative dimensions of help-seeking attitudes and mental development within the Turkish cultural context. This holistic approach enables a nuanced understanding of how these variables interact during a critical developmental period. While physical activity is widely recognized for its protective role in mental health, cultural dynamics in Turkey—such as self-stigma and fear of social judgment—may uniquely shape adolescents’ attitudes toward seeking psychological help, highlighting the importance of examining these context-specific factors. By clarifying how a modifiable lifestyle factor like physical activity relates to professional help-seeking attitudes and psychological adjustment, the study provides a theoretically grounded and culturally sensitive foundation for developing school-based preventive mental health interventions. Collectively, these contributions fill a clear empirical gap in the literature (Miles, 2017) and offer novel insights into the protective and attitudinal pathways through which physical activity may influence adolescent psychological development.

Accordingly, the general purpose of the present study is to examine the relationships among physical activity level, attitudes toward seeking psychological help, and mental development in adolescents. In line with this purpose, the following research questions were posed:

1. Is there a significant relationship between physical activity level and mental development among adolescents?
2. Is there a significant relationship between physical activity level and attitudes toward seeking psychological help?
3. Is there a significant relationship between attitudes toward seeking psychological help and mental development?

Based on previous literature, the following hypotheses were formulated:

*H1*: There is a positive and significant relationship between physical activity level and attitudes toward seeking psychological help.

*H2*: There is a positive and significant relationship between physical activity level and mental development.

*H3*: There is a positive and significant relationship between attitudes toward seeking psychological help and mental development.

## 2 Materials and methods

### 2.1 Research method

This study was designed within the quantitative research paradigm using a descriptive survey model. As Creswell (2012) emphasizes, survey research enables the systematic description and analysis of the attitudes, perceptions, behaviors, and characteristics of a target population by collecting data from the entire population or a representative sample. Accordingly, the present study employs a structured, cross-sectional survey approach to examine adolescents’ levels of psychological help-seeking attitudes and mental development in relation to their physical activity levels.

### 2.2 Participants

The sample consisted of 520 adolescents enrolled in primary and secondary schools under the jurisdiction of the Sivas Provincial Directorate of National Education. Schools were selected from a total of 108 institutions (67 middle schools and 41 high schools). To ensure adequate representation, stratified sampling was employed based on geographic distribution, socio-economic characteristics, and institutional academic achievement (Taherdoost, 2016). Specifically, schools were stratified according to socio-economic status (SES), school type, and regional distribution to ensure balanced representation across rural and urban areas. SES classification was based on neighborhood-level socio-economic profiles and administrative information provided by the Provincial Directorate of National Education. School type differentiation reflected middle and high school categories within the formal education system. To preserve geographical balance, institutions from both the city center and rural districts were proportionally included. Within each stratum, schools were selected using random procedures to enhance representativeness and minimize sampling bias. In line with these criteria, 30 schools (19 middle schools and 11 high schools) were included in the study.

Within each selected school, students were recruited through simple random sampling (Lohr, 2019). Adolescents who agreed to participate, who obtained written parental consent, and who fully completed all data collection instruments were included in the final dataset (*N* = 520). The age range of 12–18 years was adopted as an inclusion criterion for methodological reasons. Although the World Health Organization defines adolescence as spanning ages 10–19 (World Health Organization, 2025), individuals below age 12 may not fully exhibit the cognitive and psychosocial characteristics specific to adolescence. Conversely, those older than 18 are considered to be transitioning into young adulthood and thus fall outside the primary and secondary school population targeted by this study.

In addition to the predefined age range (12–18 years), a set of stringent inclusion criteria was implemented to enhance methodological rigor and internal validity. Eligibility was restricted to students who were actively enrolled in officially recognized public or private middle and high schools affiliated with the Provincial Directorate of National Education at the time of data collection. Participation was strictly voluntary, and written informed consent was obtained from all adolescents, as well as from their parents or legal guardians where applicable, in accordance with ethical standards governing research with minors. Furthermore, only participants who demonstrated adequate cognitive and linguistic competence to comprehend the survey instruments and to complete the self-report measures independently were retained in the study. To ensure data integrity, cases exhibiting substantial missing data—operationalized as more than 10% of unanswered items across the administered instruments—were systematically excluded from the final analytic sample. Additionally, individuals with temporary health conditions that could acutely affect their physical activity levels (e.g., recent injury or surgery) were excluded from participation. Detailed socio-demographic characteristics of the participants are presented in Table 1.

**Table 1:** Participant characteristics.

Variable	Categories	*n*	%
Gender	Girl	276	53.1
	Boy	244	46.9
Engagement in sports	Yes	200	38.5
	No	104	20.0
	Sometimes	216	41.5
Family support status	Yes	383	73.7
	No	36	6.9
	Sometimes	101	19.4
Physical activity level	Inactive	49	9.4
	Active	226	43.5
	Highly active	245	47.1

The sample comprised 520 adolescents (53.1% female, 46.9% male), with varying engagement in sports: 38.5% regularly participated, 41.5% participated occasionally, and 20.0% did not engage in sports. Family support was reported by the majority (73.7%), whereas 19.4% indicated occasional support and 6.9% reported no support. Physical activity levels were distributed as 9.4% inactive, 43.5% active, and 47.1% highly active, reflecting a predominantly physically engaged cohort.

### 2.3 Data collection tools

#### 2.3.1 Demographic information form

Demographic data pertaining to the participants were collected using a Demographic Information Form developed by the researchers. This form included items addressing variables such as gender, age, grade level, family income, and regular physical activity habits.

#### 2.3.2 International physical activity questionnaire-short form (IPAQ-SF)

To assess the physical activity levels of the participants, the International Physical Activity Questionnaire–Short Form (IPAQ-SF) was employed. Originally developed by Booth (2000) and subsequently refined by the International Physical Activity Assessment Group, the scale was adapted for the Turkish population through validity and reliability studies conducted by Öztürk (2005). The questionnaire consists of seven semistructured and open-ended items designed to quantitatively capture individuals’ physical activity patterns over the previous 7 days. Participants were asked to report the number of days and the average daily duration (in minutes) spent engaging in vigorous-intensity physical activity, moderate-intensity physical activity, walking, and sedentary behavior. The collected data were converted into metabolic equivalent minutes per week (MET-min/week) based on established MET coefficients: 8.0 METs for vigorous activities, 4.0 METs for moderate activities, 3.3 METs for walking, and 1.5 METs for sitting behaviors. Weekly total MET-minutes were used to categorize participants’ physical activity levels. Following the criteria proposed by Bozkuş et al. (2013), individuals with fewer than 600 MET-min/week were classified as “Inactive,” those accumulating between 600 and 3.000 MET-min/week as “Active,” and those exceeding 3.000 MET-min/week as “Highly Active.”

#### 2.3.3 Attitudes toward seeking psychological help scale (ATSPHS)

In this study, attitudes toward seeking psychological help were assessed using a revised version of the Attitudes Toward Receiving Psychological Help Scale, originally developed by Türküm (2004). The scale was subsequently revised by Karalp (2009), resulting in an 18-item instrument composed of two distinct factors. The first factor includes 12 items measuring positive attitudes toward seeking psychological help, whereas the second factor comprises six items assessing negative attitudes. The scale employs a five-point Likert-type response format, with scores ranging from 1 (strongly disagree) to 5 (strongly agree). Higher total scores indicate more favorable attitudes toward seeking psychological help. Reliability analyses conducted in the present study yielded a Cronbach’s alpha coefficient of *α* = 0.81 for the overall scale, demonstrating acceptable internal consistency. The authors of the revised version also reported a Cronbach’s alpha reliability coefficient of 0.83 for the full scale, further supporting its psychometric robustness. In the current study, the Cronbach’s alpha internal consistency coefficient for the ATSPHS was calculated as 0.728 based on the data obtained from 520 participants, indicating acceptable reliability for this Turkish adolescent sample.

#### 2.3.4 Mental development in adolescence scale (MDAS)

The Mental Development in Adolescence Scale (MDAS) was developed by Uzun (2018) to assess adolescents’ mental development levels. In the MDAS, higher scores indicate greater levels of negative developmental symptoms; therefore, elevated scores reflect increased psychological difficulties rather than more advanced mental development. Designed to identify negative symptoms specific to the adolescent developmental period, the scale consists of 15 items. Construct validity analyses revealed a two-factor structure, labeled as Personal Lack of Control and Shyness. The Personal Lack of Control factor comprises 10 items measuring symptoms such as difficulties in attention and concentration, emotional fluctuations, fear, anxiety, social withdrawal, and sudden emotional reactions. The Shyness factor includes 5 items assessing challenges related to social interaction and self-expression. The scale is administered using a five-point Likert-type response format. Factor analysis demonstrated that the two-factor structure accounted for 60.998% of the total variance. Reliability analyses yielded Cronbach’s alpha coefficients of 0.82 for the Personal Lack of Control subscale and 0.76 for the Shyness subscale, indicating strong internal consistency. The authors also reported a Cronbach’s alpha of 0.80 for the overall scale, confirming that the PDAS is a psychometrically robust and statistically reliable measurement instrument. The Cronbach’s alpha internal consistency coefficient for the Mental Development in Adolescence Scale (MDAS) was found to be 0.832 in the present study, indicating high reliability for the scale within this sample.

### 2.4 Data collection procedure

Ethical approval for the study was obtained on August 7, 2025, from the Scientific Research Proposal Ethics Assessment Committee for Social Sciences of Sivas Cumhuriyet University, and data collection was subsequently conducted between September and October 2025 during the fall semester of the 2025–2026 academic year. Following this approval, the researchers compiled a comprehensive list of all middle and high schools affiliated with the Ministry of National Education in the target province. Based on this list, schools, classes within those schools, and students within the selected classes were chosen using a random sampling procedure. The purpose of the study was communicated to students and their parents through the respective teachers. Students who voluntarily agreed to participate and whose parents provided informed consent were included in the research. The surveys were administered in a structured classroom setting within the respective school environments. Data collection was conducted in the presence of the research team, who provided standardized instructions and detailed explanations regarding the purpose of the study, voluntary participation, and confidentiality procedures prior to administration. Participants were given the opportunity to seek clarification when necessary to ensure accurate comprehension of the instruments. This supervised administration protocol was implemented to enhance procedural consistency, minimize potential misunderstandings, and reduce response bias. In the initial phase of data collection, participants completed the Personal Information Form. Subsequently, after the researchers provided the necessary instructions, the following instruments were administered sequentially: the International Physical Activity Questionnaire–Short Form (IPAQ-SF), the Attitudes Toward Seeking Psychological Help Scale (ATSPHS), and the Mental Development in Adolescence Scale (MDAS). During administration, researchers monitored whether participants experienced any physical or psychological discomfort. The data collection process lasted approximately 20 min for each school group.

### 2.5 Data analysis

All statistical analyses were conducted using IBM SPSS Statistics for Windows, Version 23.0. To examine the distributional characteristics of the variables, skewness and kurtosis values were evaluated. The results indicated the following: physical activity level (Skewness = −0.565, Kurtosis = −0.659), attitudes toward seeking psychological help (Skewness = −0.616, Kurtosis = 1.191), and mental development (Skewness = 0.171, Kurtosis = −0.314). All values fell within the acceptable range of ±1.5, suggesting that the data met the assumption of normality (Tabachnick and Fidell, 2013). Given that the normality assumption was satisfied, Pearson’s product–moment correlation coefficient (Pearson’s r)-a parametric method was employed to examine the direction and strength of linear relationships among continuous variables. Pearson’s r is a widely used and reliable statistic for evaluating the linear association between two continuous variables. For the ordinal variable (physical activity level), Spearman’s rank-order correlation coefficient was used. Correlation coefficients range from −1 to +1, with values closer to +1 indicating a strong positive relationship, values closer to −1 indicating a strong negative relationship, and values approaching zero suggesting a weak or negligible linear association (Benesty et al., 2008). In order to gain a deeper understanding of the relationships between the variables, a simple linear regression analysis was conducted. All statistical assumptions were carefully assessed, and findings were interpreted based on conventional levels of statistical significance.

## 3 Results

### 3.1 General

Prior to testing the main hypotheses, preliminary analyses were conducted to examine the internal consistency and factor structure of the measurement instruments across different physical activity levels. As presented in Tables 2, 3, within-group correlations between scale totals and their subdimensions. These correlations are expected to be high due to part-whole relationships (i.e., subdimensions contributing to total scores) and primarily serve to confirm the psychometric integrity of the scales rather than to test substantive hypotheses. The main research questions regarding the relationships between physical activity level, help-seeking attitudes, and mental development are addressed through the group comparisons presented in the following sections and the cross-scale correlations in Table 4.

**Table 2:** Results of the Spearman rank-order correlation analysis examining the relationship between adolescents’ physical activity level and their attitudes toward seeking psychological help.

Physical activity level	Coefficients	Total ATSPHS	Positive attitude	Negative attitude
Inactive (*n* = 49)	*r*	1	0.837**	0.577**
	*p*	—	< 0.001	< 0.001
Active (*n* = 226)	*r*	1	0.887**	0.482**
	*p*	—	< 0.001	< 0.001
Highly active (*n* = 245)	*r*	1	0.911**	0.552**
	*p*	—	< 0.001	< 0.001

*r*, Spearman rank-order correlation coefficient; *p*, significance value; **p < 0.01 indicates statistical significance.

**Table 3:** Results of the Spearman rank-order correlation analysis examining the relationship between adolescents’ physical activity levels and their mental development.

Physical activity level	Coefficients	Total MDAS	Personal lack of control	Shyness
Inactive (*n* = 49)	*r*	1	0.906**	0.766**
	*p*	—	< 0.001	< 0.001
Active (*n* = 226)	*r*	1	0.906**	0.649**
	*p*	—	< 0.001	< 0.001
Highly active (*n* = 245)	*r*	1	0.904**	0.626**
	*p*	—	< 0.001	< 0.001

*r*, Spearman rank-order correlation coefficient; *p*, significance value; **p < 0.01 indicates statistical significance.

**Table 4:** Pearson correlation analysis results illustrating the relationship between adolescents’ attitudes toward seeking psychological help and their mental development.

Variables	Coefficients	Total MDAS	Personal lack of control	Shyness
Total ATSPHS (*n* = 520)	*r*	0.073	0.145**	−0.041
	*p*	0.094	0.001	0.354
Positive attitude (*n* = 520)	*r*	−0.072	−0.002	−0.131**
	*p*	0.100	0.962	0.003
Negative attitude (*n* = 520)	*r*	0.290**	0.318**	0.153**
	*p*	0.001	0.001	0.001

*r*, Pearson correlation analysis; *p*, significance value; **p < 0.01 indicates statistical significance.

The physical activity levels, attitudes toward seeking psychological help, and mental development levels of the adolescents in the sample are presented in Table 5.

**Table 5:** Descriptive statistics.

Scales	Min.	Max.	Mean	Std. deviation
Physical activity level	-	-	2.37	0.65
Attitudes toward seeking psychological help				
Positive attitudes	1.00	5.00	3.01	0.75
Negative attitudes	1.00	5.00	1.92	0.68
Mental development				
Personal lack of control	1.00	5.00	2.59	0.71
Shyness	1.00	5.00	2.08	0.61

It was observed that the participants’ physical activity levels were, on average, low (𝑋̄ = 2.37, SD = 0.65). An examination of the subdimensions of attitudes toward seeking psychological help revealed that positive attitude scores (𝑋̄ = 3.01, SD = 0.75) were markedly higher than negative attitude scores (𝑋̄ = 1.92, SD = 0.68). Both positive and negative attitude scores ranged from 1.00 to 5.00, indicating notable individual differences in attitude levels among the participants. Regarding the subdimensions of the mental development scale, the mean scores for personal lack of control (𝑋̄ = 2.59, SD = 0.71) and shyness (𝑋̄ = 2.08, SD = 0.61) were below the moderate level. The minimum and maximum values (1.00–5.00) further demonstrated a wide range of variability across individuals for these constructs. Overall, while participants exhibited predominantly positive attitudes toward seeking psychological help, their physical activity levels and mental development indicators were relatively low, accompanied by substantial interindividual variability across all measured dimensions.

### 3.2 *H1*: the relationship between physical activity level and attitudes toward seeking psychological help

**Figure 1:**
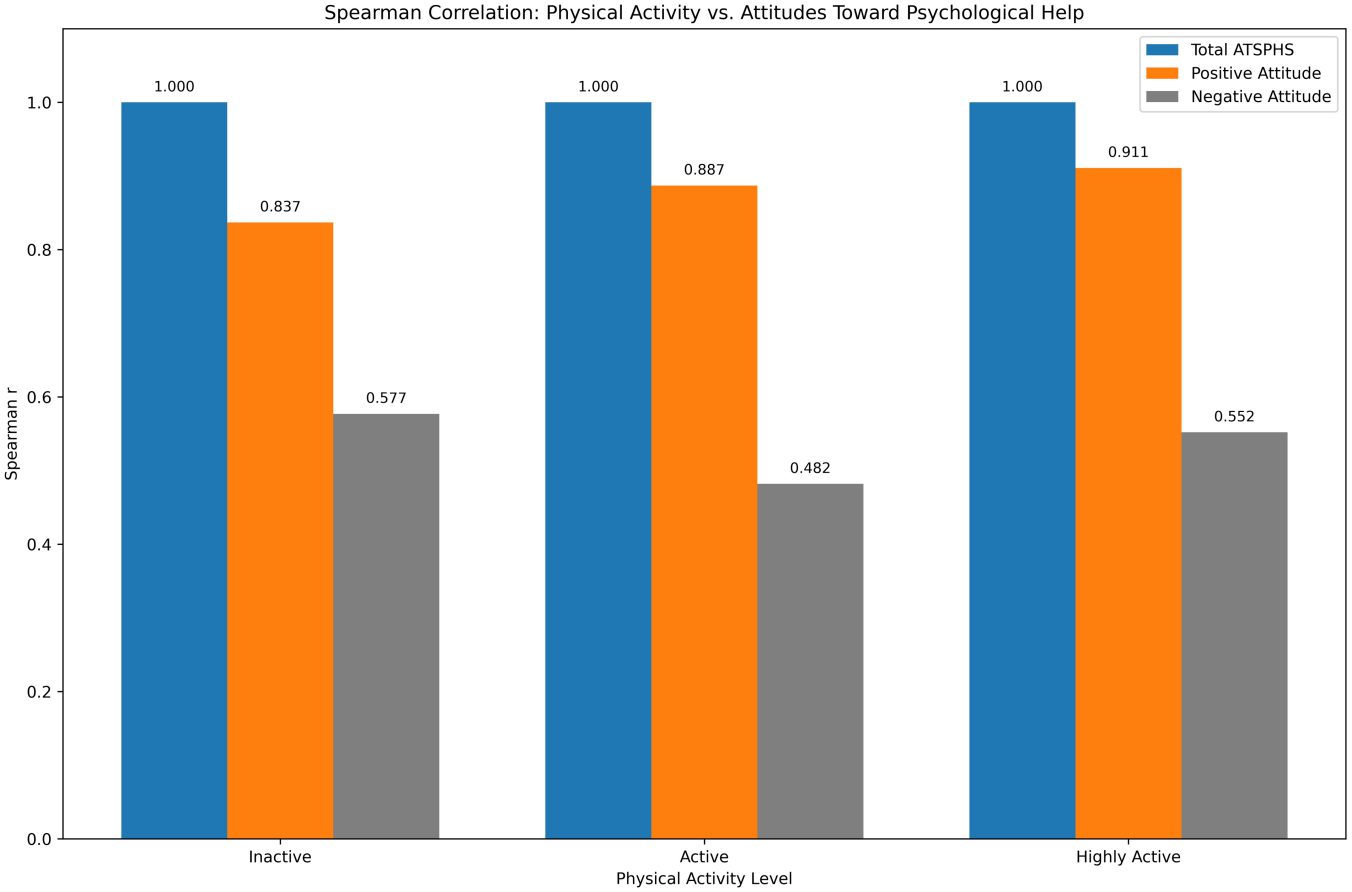
The significant association between physical activity level and attitudes toward seeking psychological help among adolescents in the sample.

The detailed results of the Spearman rank-order correlation analysis conducted to examine whether a significant association exists between the physical activity levels of the children in the sample and their attitudes toward seeking psychological help are presented in Table 2. As shown in the table, the relationships between the total attitude score and its positive and negative subdimensions are displayed across participants with inactive, active, and high levels of physical activity. As expected due to part-whole relationships, the total attitude score showed very strong positive correlations with its positive attitude subdimension across all physical activity groups (inactive: *r* = 0.837; active: *r* = 0.887; highly active: *r* = 0.911; all *p* < 0.001). Similarly, moderate positive correlations were observed between the total score and the negative attitude subdimension (*r* = 0.482–0.577, *p* < 0.001). These high correlations are anticipated because the positive attitude items contribute directly to the total score, reflecting the internal consistency of the scale rather than substantive relationships with physical activity. Similarly, a strong and positive correlation was observed in the moderately active group (*r* = 0.887, *p* < 0.001). It should be noted that these within-group correlations do not test the relationship between physical activity level and help-seeking attitudes; rather, they confirm the psychometric integrity of the measurement instrument across different activity levels. All correlations were statistically significant at the 0.001 level. This relationship is illustrated in Figure 1.

### 3.3 *H2*: relationship between physical activity level and mental development

**Figure 2:**
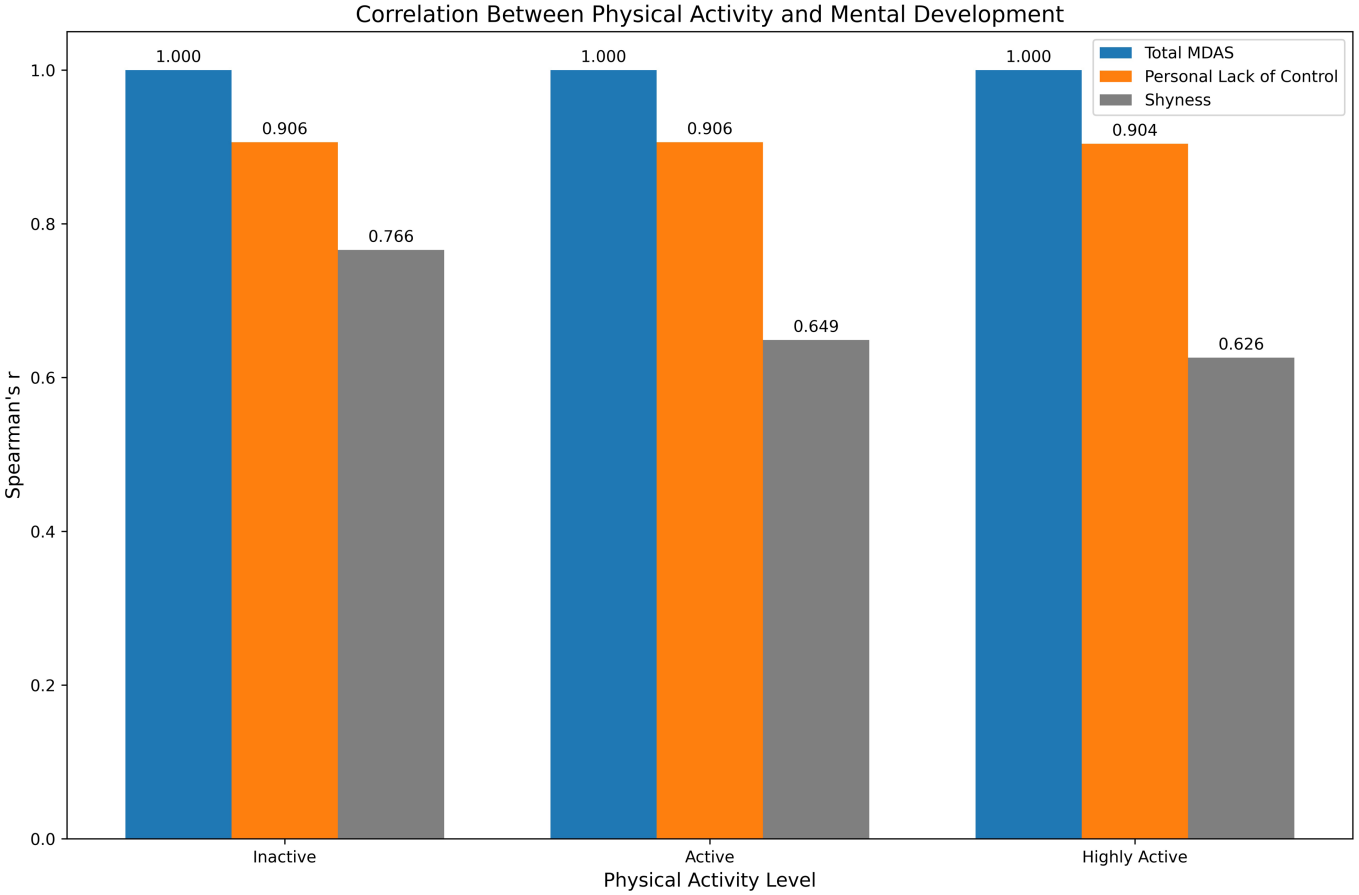
The significant association between adolescents’ physical activity levels and their mental development within the study sample.

The detailed results of the Spearman rank-order correlation analysis conducted to examine whether a significant association exists between adolescents’ physical activity levels and their mental development are presented in Table 3. Consistent with the scale’s factor structure, strong positive correlations were observed between the total mental development score and its subdimensions across all physical activity levels (*p* < 0.001). These high correlations are methodologically expected, as the subdimensions are components of the total score and share common variance. The slight variations in correlation magnitudes across groups are likely attributable to sample size differences rather than indicating a moderating effect of physical activity. These findings primarily demonstrate that the internal structure of the Mental Development in Adolescence Scale remains stable across adolescents with different physical activity levels. The nature of this association is illustrated in Figure 2.

### 3.4 *H3*: the relationship between attitudes toward seeking psychological help and mental development

**Figure 3:**
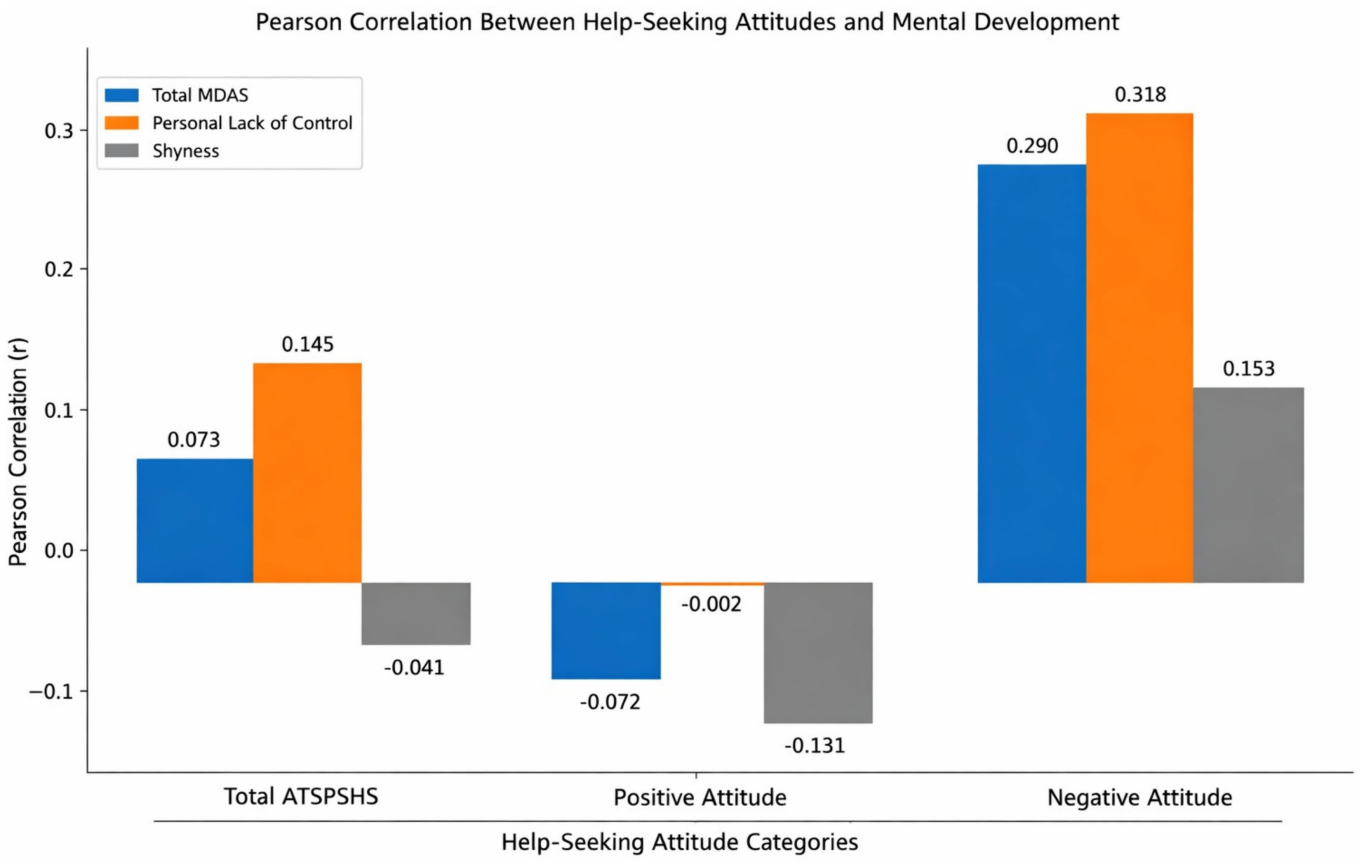
Significant relationship between adolescents’ attitudes toward seeking psychological help and their mental development in the sample.

The results of the Pearson correlation analysis conducted to examine whether there is a significant association between adolescents’ attitudes toward seeking psychological help and their mental development are presented in Table 4. As shown in the table, the correlations between help-seeking attitudes and mental development variables generally range from low to moderate in magnitude. The total score of the Attitudes Toward Seeking Psychological Help Scale (ATSPHS) was not significantly associated with the overall mental development score (*r* = 0.073, *p* = 0.094). Similarly, no significant relationship was observed between the ATSPHS total score and the Shyness subdimension (*r* = −0.041, *p* = 0.354). A weak but statistically significant positive correlation was identified between the ATSPHS total score and the Personal Lack of Control subdimension (*r* = 0.145, *p* = 0.001). Although the effect size can be classified as small according to conventional benchmarks, this finding indicates that higher perceived lack of personal control is accompanied by a slight increase in general attitudes toward seeking psychological help. Regarding the Positive Attitude subdimension, no statistically significant associations were found with total mental development (*r* = −0.072, *p* = 0.100) or with Personal Lack of Control (*r* = −0.002, *p* = 0.962). However, a weak yet statistically significant negative correlation was detected between Positive Attitude and Shyness (*r* = −0.131, *p* = 0.003). This result suggests that as shyness levels increase, positive attitudes toward seeking psychological help tend to decrease. More pronounced associations were observed for the Negative Attitude subdimension. A moderate, positive, and statistically significant correlation was found between Negative Attitude and total mental development (*r* = 0.290, *p* = 0.001). Likewise, a moderate positive and significant relationship was identified between Negative Attitude and Personal Lack of Control (*r* = 0.318, *p* = 0.001). Additionally, a weak but statistically significant positive correlation emerged between Negative Attitude and Shyness (*r* = 0.153, *p* = 0.001). Collectively, these findings indicate that as psychological maladjustment increases—particularly in terms of diminished perceived personal control—negative attitudes toward seeking professional psychological help become more pronounced. Although shyness is also positively associated with negative attitudes, the magnitude of this relationship appears comparatively weaker. Higher scores on the Mental Development in Adolescence Scale (MDAS) indicate greater psychological maladjustment, as the scale assesses negative characteristics such as lack of control and shyness. Likewise, higher scores on the Negative Attitude subdimension reflect more unfavorable help-seeking attitudes. Therefore, the observed positive correlations indicate that as psychological difficulties increase, negative attitudes toward seeking professional help also intensify. This pattern of associations is illustrated in Figure 3.

### 3.5 Simple linear regression analysis

The results of the simple linear regression analysis conducted to determine the predictive effect of physical activity level on attitudes toward seeking psychological help indicated that the model was not statistically significant [*F*(2, 517) = 2.54, *p* > 0.05]. The explanatory power of the model was very low, with physical activity level accounting for only 1% of the variance in attitudes toward seeking psychological help (R^2^ = 0.01). Examination of the regression coefficients further revealed that neither being active (*β* = 0.28, *t* = 1.80, *p* > 0.05) nor being highly active (*β* = 0.11, *t* = 0.73, *p* > 0.05) had a statistically significant predictive effect on the dependent variable (Table 6).

**Table 6:** Results of simple linear regression analysis for physical activity level predicting psychological help-seeking attitude.

Predictor	Estimate	SE	*t*	*p*	Std. estimate	95% CI	
						Lower	Upper
Intercept^a^	2.63	0.08	31.91	< 0.001			
Physical activity levels							
Active- inactive	0.16	0.09	1.81	0.072	0.28	−0.02	0.59
High active- inactive	0.07	0.09	0.73	0.463	0.11	−0.19	0.42

^a^Represents reference level.

The results of the simple linear regression analysis conducted to examine the predictive effect of physical activity level on mental development indicated that the model was statistically significant [*F*(2, 517) = 15.95, *p* < 0.001]. However, the explanatory power of the model was relatively low, with physical activity level accounting for approximately 6% of the variance in mental development (R^2^ = 0.058). Examination of the regression coefficients revealed that both being active (*β* = −0.53, *t* = −3.45, *p* < 0.001) and being highly active (*β* = −1.11, *t* = −5.36, *p* < 0.001) had a negative and statistically significant predictive effect on the dependent variable (Table 7).

**Table 7:** Results of the simple linear regression analysis examining the predictive effect of physical activity level on mental development.

Predictor	Estimate	SE	*t*	*p*	Std. estimate	95% CI	
						Lower	Upper
Intercept^a^	2.87	0.08	36.08	< 0.001			
Physical activity levels							
Active- inactive	−0.30	0.09	−3.45	< 0.001	−0.53	−0.83	−0.22
High active- inactive	−0.47	0.09	−5.36	< 0.001	−0.82	−1.11	−0.52

## 4 Discussion

The present study demonstrated that, independent of adolescents’ physical activity levels, there were strong, statistically significant, and positive correlations between the total score of attitudes toward seeking psychological help and both its positive and negative subcomponents. Similarly, robust and positive associations were identified between the total mental development score and its subdimensions across low, moderate, and high levels of physical activity. These findings indicate that adolescents’ help-seeking attitudes and mental development processes exhibit a coherent structure that appears independent of physical activity level.

The findings are partially consistent with previous literature. Turğut and Yaşar (2019) reported no significant differences between participation in sports activities and attitudes toward seeking psychological help; however, individuals who did not participate in sports demonstrated lower help-seeking attitudes than those who did. The average help-seeking attitude score in their study was 3.17, indicating a moderate openness to psychological help. These results, together with the present findings, suggest that while sport participation may contribute to psychological well-being, physical activity alone may not be sufficient to shape adolescents’ help-seeking attitudes. Supporting this interpretation, the regression analysis conducted in the present study indicated that physical activity level did not significantly predict attitudes toward seeking psychological help and explained only a very small proportion of the variance (R^2^ = 0.01), suggesting that other psychosocial factors may play a more substantial role in shaping adolescents’ help-seeking attitudes.

Previous studies conducted in Western contexts have frequently emphasized the protective role of physical activity for mental health outcomes such as depression, anxiety, and psychological well-being (Rodríguez-Romo et al., 2023; Mahindru et al., 2023). However, the absence of a direct relationship between physical activity level and help-seeking attitudes observed in this study may reflect cultural differences between Türkiye and Western societies. For instance, comparative research has shown that German and Turkish populations differ in physical activity patterns and social participation, highlighting the influence of cultural contexts on health-related behaviors (Ammar et al., 2025). While seeking professional psychological support is generally associated with lower stigma in Western societies, help-seeking decisions in the Turkish context are often shaped by family expectations, social shame, and cultural stereotypes surrounding mental health (Topkaya et al., 2017; Eruyar et al., 2024). Therefore, the psychological benefits of physical activity may not necessarily translate into more positive help-seeking attitudes in cultural environments where stigma remains a major barrier.

The strong correlations observed between mental development scores and their subdimensions across all physical activity levels further suggest that mental development may be influenced by broader psychosocial processes rather than physical activity alone. In addition, the regression analysis revealed that physical activity level significantly predicted mental development; however, the explanatory power of the model was limited (R^2^ = 0.058), indicating that physical activity accounted for only a small proportion of the variance in adolescents’ mental development.

Although physical activity is known to support children’s psychological well-being through mechanisms such as stress regulation and emotional management (Biddle and Asare, 2011; Caamaño-Navarrete et al., 2025; Biddle et al., 2019), mental development is also shaped by various contextual factors including family support, parent–child relationships, spiritual values, and academic achievement (Frankel and Hewitt, 1994; Good and Willoughby, 2006; Regnerus and Elder, 2003), as well as psychosocial skills associated with positive mental health (Savoji and Ganji, 2013). These findings suggest that adolescents’ mental development may reflect a broader ecological system of interacting individual and contextual factors.

Consistent with this perspective, help-seeking attitudes are influenced by multiple psychosocial factors, including perceived stigma, coping skills, self-efficacy, and access to social support (Barker et al., 2005). Previous studies have shown that limited family support can reduce help-seeking intentions, whereas self-stigma and public stigma represent major barriers to professional psychological support (Liddle et al., 2021; Vogel et al., 2007; Ludwikowski et al., 2009). These psychosocial barriers may help explain why physical activity levels alone were not strongly associated with help-seeking attitudes in the present study.

Another possible explanation for the absence of a direct relationship between physical activity and help-seeking attitudes may be related to characteristics of the study sample. The majority of participants were classified as active or highly active, which may have reduced variability in physical activity levels and weakened the observed associations. In addition, adolescents often perceive physical activity primarily as a social or competitive activity rather than as a strategy for improving mental health, which may limit its influence on attitudes toward professional psychological support.

Cultural factors specific to the Turkish context may also play an important role in shaping help-seeking attitudes. Research conducted with Turkish samples has consistently shown that self-stigma, masculine norms, and perceived public stigma significantly influence attitudes toward psychological help-seeking (Topkaya et al., 2017). Similarly, cross-national comparisons indicate that cultural interpretations of mental health, as well as stigma and shame, may discourage individuals from seeking professional support and instead encourage families to manage psychological distress within the family system (Eruyar et al., 2024).

Within this context, the positive relationship observed between negative help-seeking attitudes and the “Personal Lack of Control” dimension may be particularly meaningful. Adolescents who perceive a lack of personal control may experience greater feelings of shame or fear of social judgment, which could discourage them from seeking professional support. Previous research suggests that self-stigma and negative self-evaluations significantly reduce individuals’ willingness to seek psychological help (Lannin et al., 2016). In addition, evidence from child and adolescent populations indicates that perceived stigma, problem recognition, perceived benefits of help, and self-efficacy beliefs play important roles in shaping help-seeking behavior (Bandura, 1997; Radez et al., 2021).

The strong positive correlations found between negative help-seeking attitudes and mental development variables—particularly total development and Personal Lack of Control—may therefore indicate that cognitive and emotional barriers are closely linked to psychological maladjustment. During vulnerable developmental periods, reduced motivation to seek help may delay or hinder adolescents’ mental development. Conversely, the low or negative correlations observed between positive help-seeking attitudes and certain mental development dimensions suggest that help-seeking behavior may depend not only on attitudes but also on the presence of psychological need and motivation (Do et al., 2019; Abo-Rass, 2024). Adolescents’ help-seeking decisions are also influenced by their perceptions of treatment effectiveness and their trust in professionals (Rickwood and Thomas, 2012), while strong self-stigma may reduce motivation to seek help (Corrigan and Rao, 2012).

Overall, the findings of this study indicate that improving adolescents’ help-seeking attitudes and mental development requires more than simply promoting physical activity. Interventions that strengthen psychosocial skills, increase family support, and reduce stigma may be more effective in improving adolescents’ psychological adjustment and encouraging help-seeking behaviors.

## 5 Conclusion

The present findings indicate that adolescents’ help-seeking attitudes and mental development are embedded within a predominantly psychosocial framework rather than being directly shaped by physical activity levels. Although physical activity was associated with certain aspects of psychological functioning, it did not function as a decisive predictor of professional help-seeking attitudes.

The most plausible theoretical explanation is that help-seeking behavior is primarily regulated by cognitive appraisals and socio-cultural meaning systems—such as perceived self-efficacy, internalized stigma, and anticipated social evaluation—rather than by behavioral activation alone. While physical activity may enhance emotional regulation and stress management capacities, it does not inherently modify adolescents’ beliefs about vulnerability, autonomy, or social judgment.

Accordingly, strengthening adolescent mental health requires interventions that target stigma reduction, perceived control, and social support structures, in addition to promoting healthy lifestyle behaviors.

### 5.1 Limitations and future directions

This study has provided significant insights by examining the relationships among physical activity levels, attitudes toward seeking psychological help, and mental development in adolescents within a multidimensional framework. Nonetheless, several methodological and theoretical limitations should be acknowledged.

First, the cross-sectional design restricts the ability to determine the directionality and causality of the observed relationships. Consequently, future longitudinal studies are warranted to more clearly elucidate the effects of changes in physical activity and mental development on adolescents’ help-seeking attitudes. Second, data were collected via self-report measures, which are inherently susceptible to social desirability and response biases. Incorporating qualitative data collection approaches could offer a more nuanced understanding of adolescents’ help-seeking experiences. Finally, the present study focused exclusively on physical activity and did not account for other lifestyle variables, such as sleep patterns, digital media use, or nutrition. Including these factors in future research could foster a more comprehensive understanding of adolescents’ psychosocial adaptation processes.

For future research, the robust and multidimensional relationships identified in this study could be further explored using more sophisticated methodologies, including longitudinal, experimental, or mixed-methods designs. Additionally, more comprehensive theoretical models that integrate psychosocial variables should be tested, and findings should be generalized across diverse contexts. Such efforts would facilitate the development of evidence-based strategies and interventions aimed at supporting adolescents’ mental development and encouraging their engagement with psychological help services.

## Data Availability

The raw data supporting the conclusions of this article will be made available by the authors, without undue reservation.
